# Conducting a randomised trial during an influenza pandemic: an example of a trial set up and ‘hibernated’ ready to activate and recruit within 4 weeks

**DOI:** 10.1186/1745-6215-16-S2-O14

**Published:** 2015-11-16

**Authors:** Clare Brittain, Garry Meakin, Margo Childs, Lelia Duley, Wei Shen Lim

**Affiliations:** 1 Nottingham Clinical Trials Unit, Nottingham, UK; 2 Nottingham University Hospitals NHS Trust, Nottingham, UK

## Background

No randomised controlled trial has been successfully conducted during an influenza pandemic. The ASAP trial (Adjuvant Steroids in Adults with Pandemic Influenza) aims to use the novel strategy of set-up in advance of an influenza pandemic, and ‘hibernation’ in readiness for activation in a pandemic. Upon activation, the trial needs to recruit the first participant within 4 weeks, and to complete recruitment of 2200 participants within the first pandemic wave of approximately 6 weeks.

## Methods

Approvals are in place for 40 sites being maintained in ‘hibernation’. Three key programmes of work during hibernation have been identified: 1) Regular review to ensure compliance with current regulations, viability of Investigational Medicinal Product (IMP) and appropriate trial materials, 2) Planning for the ‘pre-activation’ phase (time between trial activation and first participant recruited), 3) Clinical Trials Unit (CTU) planning for activation.

## Results

Regular review includes detailed annual checks of trial site readiness and interest, review of trial materials and contracts, replacement of expiring IMP, maintenance of trial oversight committees and reporting to stakeholders. Planning for pre-activation includes packaging of IMP, developing of training materials, and logistical aspects of trial delivery including changes in key trial staff. The CTU is also developing an operational plan to support activation and reporting.

## Discussion

Long term maintenance of a clinical trial in readiness for a public health emergency is extremely challenging. Careful planning and detailed procedures should enable rapid activation and delivery of this trial. Anticipating future uncertainties is a key facet of trial maintenance during hibernation.

